# Analyzing the factors associated with efficacy among teriparatide treatment in postmenopausal women with osteoporosis

**DOI:** 10.1186/s12891-024-07227-1

**Published:** 2024-02-03

**Authors:** Meng Kong, Changtong Gao, Xiaona Luan, Cuiying Fan, Meng Hao, Canghai Jin, Jiangning Zhao, Hongyan Li, Jindong Zhao, Jian Luan, Yong Lin, Qiang Li

**Affiliations:** 1 Department of Spine Surgery, Qingdao Municipal Hospital, No. 5, Middle Dong Hai Road, Qing’dao, 266000 Shandong Province China; 2 Department of Oncology, Qingdao Municipal Hospital, No. 1, Jiao Zhou Road, Qing’dao, 266000 Shandong Province China; 3 Department of General Surgery, Ankang Hospital of Shandong Province, No. 1, Ji Dai Road, Ji’ning, 272000 Shandong Province China

**Keywords:** Teriparatide, Univariate and multivariate analyses, Response, BMD, Osteoporosis

## Abstract

**Background:**

Teriparatide (TPTD) is a widely used anabolic agent for the treatment of osteoporosis. Several factors have been identified to be related to bone mineral density (BMD) increase in anti-osteoporosis treatment with other agents; however, there has been no systematic analysis to summarize the associated determinants of BMD reaction to daily teriparatide treatment.

**Methods:**

In this retrospective study, we performed a comprehensive investigation involving not only clinical data but also several relevant lifestyle factors to be examined for their potential contribution to BMD response. This post-hoc analysis included 258 post-menopaused patients with osteoporosis who received TPTD at 20 µg/day for 12 months. Univariate and multivariate analyses were conducted to distinguish the response variables of lumbar spine (LS) BMD transformation, the principal outcome measure of efficacy, from the baseline at 12 months.

**Results:**

Twelve months of TPTD treatment resulted in an absolute 0.39 ± 0.37 increase in T-score of LS BMD. Gastrointestinal disease, prior bisphosphonate or glucocorticoid treatment, no vitamin K2 supplementation, low levels of serum 25(OH)D and PINP, weak increment of PINP and β-CTX at 3 months, unhealthy lifestyle (excessive smoking, tea, coffee, and drinking), vegetarian diet pattern, low ALT level, and high BMD at baseline were determined by univariate analyses to be related to the weak reaction of TPTD treatment (*P* < 0.10). In the multiple regression model, postmenopausal women with vitamin K2 supplementation, higher baseline serum 25(OH)D level, and higher PINP concentration at 3 months indicated a good reaction of LS BMD at 12 months (*P* < 0.05). Patients with gastrointestinal disease, prior bisphosphonate and glucocorticoid treatment, vegetarian diet pattern, and higher baseline BMD were significantly more likely to have a lower absolute LS BMD response compared to patients without these characteristics (*P* < 0.05). Further analysis confirmed the negative effect of unhealthy lifestyle on TPTD treatment.

**Conclusion:**

Our results emphasize the significance of a comprehensive assessment of clinical or lifestyle-related characteristics of postmenopausal women with osteoporosis in the management of TPTD therapy in routine care.

**Supplementary Information:**

The online version contains supplementary material available at 10.1186/s12891-024-07227-1.

## Introduction

Osteoporosis is characterized by decreased bone mass and impaired bone microarchitecture, and is the most prevalent skeletal disorder of bone metabolism. As a chronic and systemic degenerative disease, the mechanism of osteoporosis is the disequilibrium between the bone resorption capacity of osteoclasts and bone formation capacity of osteoblasts [1]. Numerous studies have suggested the combined and long-term management of osteoporosis [2]. Different treatments for osteoporosis are available in clinical studies. Various options are consistently aimed at maintaining bone health and decreasing the risk of fracture. Currently, approved agents for the treatment of osteoporosis can be classified as antiresorptive or osteoanabolic agents. The current anti-osteoporosis therapeutic strategies can be divided into two categories: anti-bone resorption and osteoanabolism.

The effects of anti-osteoporosis treatments can be influenced by several factors. According to previous studies, baseline and early serum bone turnover markers (BTMs) transformation might affect bone mineral density (BMD) response in women who received anti-osteoporosis therapies [3, 4]. In addition, it has been postulated that a patient’s response to osteoporosis therapy may be affected by insufficient vitamin D level [5]. In addition, lifestyle, dietary habits, smoking, physical inactivity, and serum estradiol levels have been found to be related to the effects of treatment [6]. However, some medicines may have adverse effects on bone health and induce secondary osteoporosis in adults, such as glucocorticoids, which may cause negative effects during the course of anti-osteoporosis treatment [7].

Teriparatide (TPTD, recombinant human PTH(1–34)) is a widely used anabolic agent for osteoporosis, which acts by means of increasing bone turnover by the classic remodeling cycle involving both osteoclastic resorption and osteoblastic reformation and stimulating new bone formation on otherwise quiescent bone surfaces [8]. Considering its definite curative effect in the treatment of postmenopausal women with severe osteoporosis and high risk for fractures, TPTD was recommended as one of the first-line drugs. Qingdao City, Shandong Province, has been a unique region covering this medicine under Health Insurance in China since 2013. For these reasons, thousands of postmenopausal women with osteoporosis have received the TPTD injections prescribed by our medical team. Overall, we have defined the effectiveness of this medicine on lumbar spine BMD improvement, reducing the incidence of vertebral compression fractures (VCFs) and back pain, and improving the quality of life [9]. However, during treatment with teriparatide at 20 µg/day, not all patients had a positive outcome in BMD, and some subjects responded less well to this agent, suggesting the inconsistent potency of this therapeutic regimen in different individuals.

Thus, in this study, we evaluated many potential factors that might be associated with the efficacy of teriparatide treatment in postmenopausal women with osteoporosis, using retrospective clinical information collected from previous drug recipients. Hoping that the information found here might be useful to healthcare providers assessing optimal TPTD users whose properties are suitable, or controlling unfavorable factors to maximize the effect when TPTD is administered in routine care. Although many factors identified in this study, such as BMD and PINP levels at baseline, have already been predicted to be associated with absolute BMD gain after teriparatide treatment, no previous study has considered multiple clinical and lifestyle factors and conducted a comprehensive analysis.

## Materials and methods

### Study design and subjects

This retrospective study included patients treated with TPTD in our department. All the data were collected from a regular registry. A computerized database was searched to identify standard candidates who met the following inclusion criteria:1) female patients at least 2 years post-menopause with primary osteoporosis or who suffered from glucocorticoid-induced osteoporosis; 2) serum creatinine level < 2 mg/dl, with specific requirements including normal excretion of serum calcium, endogenous parathyroid hormone, and urinary calcium; 3) patients who completed the 12-month TPTD treatment without interruption and were able to complete drug injection; and 4) in addition to osteoporosis, freedom from severe or chronically disabling conditions.

The exclusion criteria were as follows:1) treatment course of an endocrine disorder (in addition to type 2 diabetes) or aromatase inhibitors; 2) malignant neoplasms, multiple myeloma, and history of diseases other than postmenopausal osteoporosis that affect bone metabolism and drug abuse.

TPTD was administered at a dose of 20 µg daily by subcutaneous self-injection, together with other adjuvant drugs supplemented with calcium (600 mg/d) and vitamin D (1200–2000 IU/d), with or without vitamin K2 (Menatetrenone Soft Capsules, 45 mg/day; Business Guide-Sha, Japan). Prior use of any antiresorptive (AR) agents (such as bisphosphonates, raloxifene, estrogens, and estrogen/progestin) was allowed without limitation or washout periods; however, these drugs had to be discontinued at baseline. The study was conducted between January 2018 and April 2022. Parameters that may be associated with osteoporosis were assessed as follows:

### Assessment of blood data

At baseline, venous blood sampling was performed to assess the following eight items: glutamic oxaloacetic transaminase (AST), glutamic pyruvate transaminase (ALT), total cholesterol (TC), uric acid (UA), creatinine, 25(OH)Vit D, and two serum biochemical bone markers (type I procollagen N-terminal pro-peptide (P1NP) and type I collagen cross-linked C-telopeptide (β-CTX), which were repeatedly measured at 3 months percentage change from baseline to 3 months).

In particular, it should be mentioned that BTMs (25(OH)Vit D, P1NP, β-CTX) were all measured using electrochemiluminescence assay (ELECSYS, Roche Diagnostics GmbH, Mannheim, Germany). The intra- and inter-assay coefficients of variation (CV) were: 4.3–6.8% and 3.9 − 5.9%, respectively, for 25(OH)Vit D; 4.0–6.1% and 4.6–7.0% for P1NP; 5.5–7.6% and 6.5–8.8% for β-CTX.

### Lifestyle factors

Single questions were asked to evaluate the lifestyle factors involved in smoking and tea, coffee, and alcohol consumption.

Women who smoked regularly every day or had discontinued smoking were defined as current or past smokers, and those who never smoked were defined as never smokers.

People who consumed more than 2 cups (200 ml/d) of tea were defined as habitual drinkers, and those who had no tea drinking habit or drank 1 or 2 cups every day were classified as non-drinkers or not regular drinkers. Women who regularly consumed more than two cups (200 ml) per day were classified as habitual drinkers, and women who never drank coffee or drank one or two cups per day were classified as non- or non-regular drinkers. Consumption of one cup (100 mL) of coffee corresponds to an intake of 50 mg caffeine [10, 11]. Alcohol consumption was determined using self-administered questionnaires and classified into four groups: non-drinkers (0 g/day), light drinking (1–9 g/day women), moderate drinking (10–29 g/day women), and heavy drinkers (≥ 20 g/day women) [12].

During previous routine treatment practice, we used a modified version of the original food frequency questionnaire (FFQ) [13] called the Internet-based Food Frequency Questionnaire for Chinese (IDQC) for dietary assessment, which was developed by Feng et al. to investigate the types, frequency, and intake of the surveyed residents over the past 12 months based on the dietary characteristics of local residents [14]. The IDQC comprises 136 food items commonly consumed by Chinese people. Participants answered how frequently they consumed each of the 136 food items by choosing one of the following eight options: almost null, one–three times a month, once a week, two–three times a week, four–five times a week, once daily, twice daily, thrice daily, and more. Regarding dietary habits, using the answers to IDQC, we extracted four common dietary patterns: Mediterranean Diet, Western Diet (high-fat), Asian Diet (plant-based dietary) and Vegetarian Diet [15, 16].

With respect to physical activities (PA), participants answered questions about time and frequency spent on daily activities in the Chinese version of the New Zealand Physical Activity Questionnaire-Short Form (NZPAQ-SF) [17, 18]. We used MET-min/day to quantify physical activity, which was calculated using the scoring rule of the International Physical Activity Questionnaire (IPAQ) for continuous scores. The MET values and the numerical procedure for MET-min are as follows [19]:

Walking MET-min/week at work = 3.3 * walking minutes × walking days at work.

Moderate MET-min/week at work = 4.0 × moderate-intensity activity minutes × moderate-intensity days at work.

Vigorous MET minutes/week at work = 8.0 × vigorous-intensity activity minutes × vigorous intensity days at work.

Total Work MET-minutes/week = sum of walking + moderate + vigorous MET-minutes/week scores at work.

Total PA MET-minutes/week = sum of walking + moderate + vigorous MET-minutes/week.

According to the physical activity evaluation criteria, the participants were categorized into four levels: sedentary, adequately activated, adequately activated, and adequately activated with strenuous exercise [17].

### Demographic data

Demographic and baseline characteristics (age, age at menopause, and BMI), prior hypertension, diabetes, gastrointestinal disease, prior bisphosphonate use, and glucocorticoid treatment were summarized using descriptive statistics.

### Clinical outcomes measurements

For clinical outcome measurement, BMD in the lumbar spine and hip were measured using the same dual-energy X-ray absorptiometry (DXA) (PIXImus2; Lunar, GE, USA); vertebrae were excluded if percutaneous vertebral augmentation or internal fixation was performed when calculating the T-values.

For derived change values, participants with both a baseline value and at least one post-baseline value of a measurement were included in the analysis.

### Statistical analyses

We performed a simple regression analysis to examine the relationship between the absolute changes in BMD and different variable terms. Changes in BMD as the enumeration response variable and prognostic factors were used as the regression variables. We then extracted items that were potentially associated with the effect of anti-osteoporosis treatment using a criterion of *P* < 0.10 to avoid overfitting of the model. After identifying these factors as explanatory variables and BMD change values as dependent variables, we performed multiple regression analysis. Statistical significance was set at *P* < 0.05.

Continuous variables are expressed as mean values with standard deviations [SD]. All statistical analyses were performed using R programming language (version 4.2.1; R Foundation).

## Results

### Characteristics of patients

Age, age at menopause, BMI, prior osteoporotic fractures, prior gastrointestinal disease, hypertension and diabetes, prior bisphosphonate and glucocorticoid treatment, vitamin K2 supplementation, serum levels of ALT, AST, TC, UA, creatinine, 25(OH)D, PINP, and β-CTX concentrations; lumbar spine (LS) BMD at baseline; lifestyle factors, dietary patterns, and physical activities are reported in Table 1. The normal reference intervals for necessary serum markers involved were showed in Supplementary form.

**Table 1:** Characteristics of the study subjects (*n* = 258)

Variable	Mean (SD), n	Variable		Mean (SD), n
Age (years)	70.05 ± 8.95	Smoking (0/1/2^a^)		243/13/2
Age of menopause (years)	50.44 ± 2.22	Tea (0/1/2^a^)		243/15/0
BMI (kg/m^2^)	24.00 ± 3.07	Coffee (0/1/2^a^)		246/11/1
Previous osteoporosis fracture (yes/no)	174/84	Alcohol (0/1/2/3^a^)		243/14/1/0
Gastrointestinal disease (yes/no)	24/234	Diet pattern	Mediterranean	76
Hypertension (yes/no)	80/178		Western	57
Diabetes (yes/no)	31/227		Asian	96
Bisphosphonates (yes/no)	15/243		Vegetarian	29
Glucocorticoid (yes/no)	28/230	Exercise (1/2/3/4^b^)		37/143/67/11
Menatetrenone/Vit K2 (yes/no)	32/226	ALT (U/L)		26.25 ± 11.76
25(OH)D level at baseline (ng/ml)	20.33 ± 7.18	AST (U/L)		28.78 ± 10
PINP level at baseline (ng/ml)	63.46 ± 22.66	TG (mmol/L)		4.72 ± 1.1
β-CTX level at baseline(ng/ml)	0.65 ± 0.26	UA (umol/L)		228.88 ± 80.4
PINP increment (3 month)	74.75 ± 54.90	Creatine (umol/L)		72.27 ± 17
β-CTX increment (3 month)	0.22 ± 0.31	LS BMD at baseline		-3.3 ± 0.93

*LS* Lumbar spine

^a^Assessment criteria was detailed in Table 2

^b^Respectively represent Sedentary, In-sufficiently active, Sufficiently active, Sufficiently active with vigorous intensity activity

### Changes in BMD in response to teriparatide treatment

Twelve months of teriparatide therapy resulted in a positive BMD response in the T-scores of the LS and Ward BMD. The average absolute elevation in T-score of LS BMD was 0.39 ± 0.37, and total hip BMD increase was 0.09 ± 0.31 (data not shown). Finally, we focused on the following analysis of the LS BMD response, considering the weak reaction of Ward BMD.

### Identification of the potential factors associated with BMD response evaluated by simple regression analysis

The effects of clinical data and follow-up index on LS BMD elevation were evaluated using univariate analysis. As shown in Table 2, we identified 13 baseline factors that were potentially associated with lower BMD response (*P* < 0.10): gastrointestinal disease, prior bisphosphonate or glucocorticoid treatment, no vitamin K2 supplementation, low serum 25(OH)D level, low serum PINP level, unhealthy lifestyle (including excessive smoking, tea, coffee, and drinking), vegetarian diet pattern, low ALT level, and high BMD at baseline. As follow-up factors, low elevated fluctuations in PINP and β-CTX at 3 months were also related to feeble reactions (*P* < 0.10).

**Table 2:** Univariate analyses between different variables and LS BMD change at 12 months

Parameter	Absolute change		Parameter	Absolute change	
	Estimate	*P* value		Estimate	*P* value
Age	0.001945	0.451	Smoking	-0.28717	0.000461***
Age of menopause	-0.01268	0.2181	Tea	-0.20749	0.0343*
BMI	-0.005369	0.47283	Coffee	-0.18500	0.0574
Previous osteoporosis fracture	0.009811	0.842	Alcohol	-0.20109	0.0243*
Gastrointestinal disease	-0.22190	0.0048**	Western Diet^a^	-0.0008772	0.98900
Hypertension	0.01267	0.799	Asian Diet^a^	-0.0446272	0.42381
Diabetes	-0.07522	0.288	Vegetarian Diet^a^	-0.2549002	0.00145**
Bisphosphonates	-0.27827	0.00438**	Exercise	-0.03574	0.257
Glucocorticoid	-0.21174	0.00395**	ALT	-0.003383	0.0832
Menatetrenone/Vit K2	0.14386	0.0217*	AST	-0.001340	0.55
25(OH)D level at baseline	0.015260	4.95e-07***	TG	-0.01010	0.617
PINP level at baseline	0.0017412	0.0804	UA	9.443e-05	0.742
β-CTX level at baseline	0.09458	0.27	Creatine	-0.001141	0.4
PINP increment (3 month)	0.0027323	9.76e-12***	BMD at baseline	-0.13610	1.45e-08***
β-CTX increment (3 month)	0.21086	0.00451**			

^a^Estimate was compared with Mediterranean Diet

### Identification of the determinants associated with BMD response evaluated by multivariate regression analysis

**Fig. 1:**
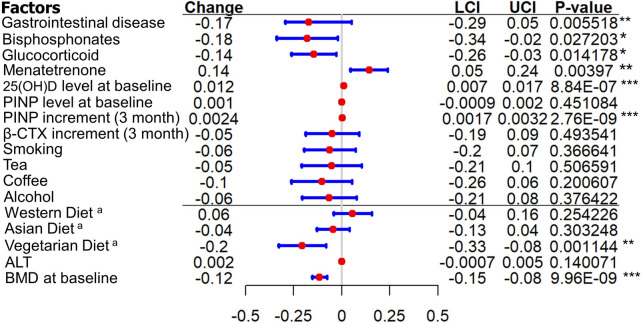
Forest plot of multivariate-analysis showing the effect of different items on lumbar spine BMD response to TPTD treatment. LCI, lower confidence interval. UCI, upper confidence interval. BMD and bone mineral density. ^a^Estimate was compared with Mediterranean Diet

The data were further analyzed using multivariate analysis. The results are presented in Table 3; Fig. 1. In the multiple regression model (which included the 15 aforementioned potential factors), patients with gastrointestinal disease, prior bisphosphonate and glucocorticoid treatment, vegetarian diet pattern, and higher baseline BMD were significantly more likely to have a lower absolute LS BMD response than those without these characteristics (*P* < 0.05). Furthermore, vitamin K2 supplementation, higher serum 25(OH)D levels at baseline, and higher PINP concentration increase at 3 months indicated a good reaction of LS BMD at 12 months (*P* < 0.05). As there was no significant relationship between the single aspect of lifestyle and LS BMD change during the TPTD therapeutic course, a further multiple regression analysis involving comprehensive elements based on the four sub-items (smoking, tea, coffee, and drinking) was conducted. The frequencies and amplitudes of the response scales were measured at an ordinal level of 0 to 3 points. The total lifestyle score was obtained by summing the scores for the four living habits (Table 4), and the positive relationship between a healthy lifestyle and TPTD treatment response was confirmed. Estimate (r) = -0.0709, 95%CI -0.1346 - (-0.0071), *P*<0.05.

**Table 3:** Multivariate analyses between different variables and LS BMD change at 12 months

Risk factors	Absolute change		
	Estimate (r)	95%CI	*P* value
Gastrointestinal disease	-0.1721034	-0.2931635538 - (-0.051043176)	0.005518**
Bisphosphonates	-0.1795748	-0.3387616315 - (-0.020387906)	0.027203*
Glucocorticoid	-0.1449302	-0.2604801397 - (-0.029380199)	0.014178*
Menatetrenone	0.1427399	0.0460707088–0.239409133	0.003970**
25(OH)D level at baseline	0.0122580	0.0074742986–0.017041711	8.84e-07***
PINP level at baseline	0.0005970	-0.0009609306–0.002154868	0.451084
PINP increment (3 month)	0.0024547	0.0016720266–0.003237379	2.76e-09***
β-CTX increment (3 month)	-0.0484735	-0.1877221096–0.090775017	0.493541
Smoking	-0.0616644	-0.195963231–0.0726345088	0.366641
Tea	-0.0525861	-0.208323190–0.1031510294	0.506591
Coffee	-0.1030199	-0.261151875–0.0551119760	0.200607
Alcohol	-0.0639997	-0.206269747–0.0782702642	0.376422
Western Diet^a^	0.0573259	-0.0414822115–0.156134037	0.254226
Asian Diet^a^	-0.0444775	-0.1294011073–0.040446011	0.303248
Vegetarian Diet^a^	-0.2043182	-0.3265763246 - (-0.082060024)	0.001144**
ALT	0.0023003	-0.0007605353–0.005361098	0.140071
BMD at baseline	-0.1151999	-0.1534059432 - (-0.076993822)	9.96e-09***

^a^Estimate was compared with Mediterranean Diet

**Table 4:** Condensed version of the questions evaluated, and response alternatives of daily life

Question	Response alternatives
A. General cigarettes intake in engaged work or daily life	0. Never smoked
	1. Quit smoking
	2. Regularly, everyday
B. General tea intake in engaged work or daily life	0. Never drank tea
	1. 1 or 2 cups per day
	2. Regularly, more than 2 cups (200 ml) per day
C. Coffee consumption in daily life	0. Never drank coffee
	1. 1 or 2 cups per day
	2. Regularly, more than 2 cups (200 ml) per day
D. Alcohol consumption in daily life	0. Non-drinkers (0 g/day)
	1. Light drinking (1–9 g/day women)
	2. Moderate drinking (10–29 g/day women)
	3. Heavy drinking (≥ 20 g/day women)

Each option was given equal weight in comprehensive score

## Discussion

Serial bone mineral density (BMD) measurements are currently used to assess response to osteoporosis treatment. This study investigated the association between various baseline characteristics, lifestyle factors, and lumbar BMD elevations in response to pharmacotherapy. To our knowledge, the association between multidimensional clinical determinants and therapeutic effects of TPTD treatment has not been previously studied.

Based on the current results, BMD could increase with teriparatide treatment; however, the range of the reaction was inconsistent. In models fully adjusted for other variables, with higher baseline levels and amplitude of variation in BTMs, the percentage change in BMD tended to be greater. Several studies have demonstrated that bone turnover status at baseline might correlate with subsequent BMD responses to treatment [20]. As the production of type I procollagen is converted into mature type I collagen, the usefulness of PINP levels during daily teriparatide treatment has been frequently reported. Niimi et al. [21] examined the baseline characteristics of lower PINP concentrations and found that lower early increases in PINP were significantly associated with a blunted LS bone BMD response following teriparatide treatment. Consequently, serum PINP monitoring might be useful clinically and in clinical trials and is considered an important marker for monitoring patients receiving teriparatide [22].

From another perspective, the rate of formation of bone remodeling units would, interestingly, double after menopause, among which the amount of new bone is reduced compared with that which is newly resorbed in the same remodeling cycle. That, in fact, signifies remodeling imbalance and underlie the accelerated bone loss and osteoporosis [23]. Increased concentrations of BTMs, can be associated with increased rates of bone loss [24, 25]. At the molecular level, high BTM transformation, especially bone resorption markers such as CTX-I, indicates that more reconstruction exists on the bone surface and contains more osteoid tissue (not yet mineralized bone) than in normal circumstances [26]. Hence, in the current study, the correlation between variation in bone formation markers and spinal BMD response could be ascribed to highly effective bony reconstitution and greater teriparatide responsiveness in the bone trabecula region. Since bone markers quickly respond to changes in bone physiology and occur earlier than variations in BMD, monitoring of baseline status and changes in BTMs induced by TPTD play an important role in characterizing pharmacotherapy effects on the basic multicellular units and would be informative for clinicians during the management of teriparatide treatment for osteoporosis patients. Of particular note is that because bone strength largely represents the promotion of a series of indicators involving bone microstructure, composition, and BMD, it would seem more rational to add biochemical markers as an additional objective index in the evaluation of the therapeutic efficiency of teriparatide.

Clinically, most antiresorptive agents inhibit bone resorption, resulting in decreased bone remodeling (aimed at restoring remodeling balance). A decrease in bone resorption subsequently results in a rapid reduction in bone formation because of the reduced release of the coupling factors [27]. As previously described, the duration of antiresorptive treatment is negatively associated with BMD changes in the lumbar spine in a multifaceted model [28]. Teriparatide increases bone remodeling—both bone formation and bone resorption—resulting in a net gain of new bone, but effects of TPTD might be slightly blunted, it would consume about 6 months, be called an ‘anabolic window’ [29], that teriparatide during the initial treatment phase overcomes the inhibition of bone remodeling induced by prior antiresorptive therapy [30, 31]. In this study, data on prior osteoporosis treatments were obtained retrospectively at baseline; however, we did not have accurate details regarding adherence and compliance to these treatments. Hence, it might consume the necessary time for the reabsorption of extended calcified bone induced by long-term pre-treatment [32].

Numerous epidemiological studies have evaluated the ubiquity of serum 25(OH)D insufficiency, which is a common issue worldwide [5]. Several credible data have confirmed that the addition of vitamin D and calcium to anti-osteoporotic treatment is necessary unless the patient is vitamin D replete. It has been postulated that vitamin D insufficiency may affect a patient’s response to osteoporosis therapy, and the status of vitamin D insufficiency could affect the percentage change in LS BMD after 12 months of treatment [26]. Consistently, we found that low level of 25 hydroxyvitamin D at baseline might be a negative factor during the course of TPTD treatment, which could be more obvious when the level was < 10 ng/ml. An interesting finding in the current investigation is that a low 25(OH)D level was associated with a weak BMD response independent of BTMs changes, which suggests that vitamin D deficiency might impair TPTD responses by mechanisms such as negatively influencing bone mineralization rather than by interfering with bone turnover, for instance, by inducing secondary hyperparathyroidism. Further research on the co-administration of vitamin D supplements (form, dosage, and course) is important.

Vit K acts as a cofactor for carboxylases. This facilitates the gamma-carboxylation of osteocalcin (OC), a non-collagenous protein with high content in the bone, and allows the protein with high affinity for calcium ions to bind to osteocalcin and hydroxyapatite, thus promoting osteogenesis [33, 34]. Studies have demonstrated that if the process of gamma-carboxylation is hindered by a lack of vitamin K, the concentration of a lower hydroxyapatite affined molecule, undercarboxylated osteocalcin (UcOC), would be upregulated [35], which is inversely related to BMD and might make postmenopausal women vulnerable to bone fractures [34]. Other data suggest that vitamin K2 may enhance bone healing by promoting the differentiation of osteoblasts through steroids and xenobiotic receptors (SXR) [36, 37]. in vivo experiments have reported that impaired γ-carboxylation of OC, induced by vitamin K insufficiency, could attenuate the enhancing effect of PTH1–34 therapy on the biomechanical recovery of osteotomized bone in rats, whereas combined treatment with Vit K2 and TPTD may be more effective than monotherapy for postmenopausal osteoporosis, perhaps through the increase in OC and the activation of Ob.S (the number of osteoblasts attached to the surface of the cancellous bone) [38], which was verified in our results. Nonetheless, because of the lack of an accurate reference value exhibiting a normal level, the assessment of the Vit K2 status remains a matter of discussion, which would restrict its practical use in medicine. According to some studies, the calculation of the ucOC/cOC ratio might be considered an indirect parameter for obtaining information about the Vit K2 status [39]. Further investigation is needed to explore the efficacy of vitamin K2 replenishment at physiological and pharmacological doses, and the appropriate dose of vitamin K2 to ensure the best effect of TPTD.

A low BMD is a well-established risk factor for osteoporotic fractures. However, several academics have suggested that anti-osteoporotic pharmacotherapy is less effective in patients with relatively higher BMD [40, 41]. This study showed consistent results of negative relationship between basal BMD and therapeutic response during TPTD treatment with previous studies, in which no conflicting conclusions were derived although various typical anti-osteoporotic medications, including alendronate, risedronate, raloxifene and teriparatide were involved [41–43].

Chronic glucocorticoid (GC) therapy has also been associated with osteoporosis (GIO). Several studies have demonstrated that daily doses of prednisone as low as 10 mg may result in a significant clinical loss of bone mass [44]. The pathological characteristics of GIO are primarily chronically disturbed or suppressed bone formation, as GCs reduce osteoblast function and replication as well as increase osteoblast apoptosis [45], simultaneously accompanied by bone resorption elevation in the initial period (the first 12 months of the treatment program) by lengthening the lifespan of osteoclasts [46]. As described for the pathogenesis of GIO, bone anabolic agents may offer a preferred management option. Previous randomized, double-blind trial demonstrated the certain statistically significantly advantages, including increments in BMD and reduction in new vertebral fractures incidence, of daily subcutaneous injections of teriparatide (20 µg) in patients with GIO compared with bisphosphonates [47, 48]. Primary clinical trial analysis suggested that excessive long-term glucocorticoid use attenuates the LS BMD response to TPTD relative to lower doses, which might be due to the primary opposing actions of TPTD and glucocorticoids directly on osteoblasts [49]. Therefore, according to the 2016 AACE (American Association of Clinical Endocrinologists) and ACE (American College of Endocrinology) Clinical Practice Guidelines, teriparatide can be considered an alternative first-line option in patients with the highest fracture risk, for instance, long-term administration of GC and BMD T<-3.0 [50].

Regarding the influence of history of fracture on anti-osteoporosis therapeutic effects, some scholars have previously conducted early studies. Rossini [51] and Carr et al. [52] demonstrated that adherence to treatment was significantly higher in patients with previous vertebral fractures, since a history of fractures would improve motivation to use prescribed medication; in particular, the presence of persistent pain after fracture would be the determining factor in decreasing the risk of treatment discontinuity. Interestingly, another study concluded that a family history of hip fracture is also associated with increased persistence of treatment with anti-osteoporosis drugs [53]. However, our results showed no statistical correlation between prior osteoporotic fractures and subsequent increase in LS BMD. Considering economics, subjects were less likely to unrespect the prescription because of the high cost of medicine.

Several studies have aimed to determine whether combined pharmaceutical/loading therapies are more effective than either treatment alone [54]. This idea stems from the belief that if bones are not energized and physically active, mechanoreceptors (osteocytes) do not receive signals regarding the need for remodeling, removal of damaged bone, and synthesis of new bone; thus, there is a gradual reduction in total bone mineral density [55]. As mechanical loading has been shown to promote bone turnover-favoring formation, a combination of pharmaceutical treatment and exercise loading may have an additive effect on bone health. As the National Osteoporosis Foundation has demonstrated, active walking is one of the most effective forms of exercise for the maintenance or improvement of bone mineral density in postmenopausal women. In addition, it has been reported that not only exercises by load of its own weight or exercise against resistance are effective for increasing bone density, but aerobic exercises also increase the balance and functional activity of muscles, thus reducing the risk of falls [56]. However, a definitive conclusion regarding the combined effects of pharmaceuticals and exercise loading requires better methods to measure and monitor loading, as no obvious relationship was found.

Osteoporosis therapy typically includes not only the employment of pharmacological agents, but research has also suggested that lifestyle interventions such as physical activity and modifiable lifestyle factors (smoking, tea, coffee, drinking et al.) both play important roles in bone health [57, 58]. Although the positive role of some bone-promoting lifestyle factors was not confirmed favorably in this study and lifestyle modifications alone may not be adequate to improve bone quality, which does not mean that they do not generally occur in the population, they form an important basis before initiating pharmacological approaches to prevent or treat osteoporosis. According to this research, several unhealthy habits may cause negative cumulative dose effects on treatment, which means that lifestyle change advice is still necessary and is a key adjuvant treatment. Moreover, protein intake, particularly an animal protein diet, is known to provide the necessary ingredients associated with bone transformation [59]. Similarly, in this study, a vegetarian diet was significantly associated with a lower BMD increase, suggesting that nutritional imbalance is an unfavorable factor for anti-osteoporosis therapy. We believe that supplementation with the certain nutrients deficient in a vegetarian diet would contribute effectively to the curative response of TPTD, r and further experiments and data are required. To some extent, the efficient uptake and utilization of several bone metabolite-related elements, such as calcium, magnesium, and phosphorus, can also be disrupted by many gastrointestinal diseases, following conditions: Crohn’s disease, ulcerative colitis, celiac disease, post-gastrectomy, short bowel syndrome, chronic hepatitis, and cirrhosis et al. [60]. In addition, among these patients, the widespread use of proton pump inhibitors (PPIs) has been recognized as potentially linked to osteoporosis and bone loss, as well as a weakened response [61], which was mentioned by the FDA in 2010 regarding the potential fracture risk (http://www.fda.gov/Drugs/DrugSafety/PostmarketDrugSafetyInformationforPatientsa.

ndProviders/ucm213206.htm). Therefore, it is no exaggeration that all postmenopausal women, regardless of BMD and pharmacotherapy methods, should be counseled to eat a balanced diet, exercise regularly, avoid smoking or excess alcohol use, and reduce tea or coffee consumption, and follow recommendation to prevent falls [58, 62].

To the best of our knowledge, no comprehensive analysis evaluating BMD response to daily teriparatide treatment involving multiple determinants has been conducted. The present study demonstrated that low serum 25(OH)D levels, higher BMD and low PINP levels at baseline, or minor PINP elevation at 6 months were independently associated with poorer efficacy during TPTD treatment in postmenopausal subjects with osteoporosis. The identification of these factors and pertinent management of such patients would assist in the treatment strategy, such as correction of vitamin D deficiency, vitamin K2 supplementation, establishing a regular healthy lifestyle, and enforcement of drug adherence. We recommend that physicians take these lines of informative evidence into consideration in clinical practice to select potential optimal TPTD responders whose properties are suitable, make more precise treatment strategies, and improve the utilization of daily teriparatide.

### Limitation

As all subjects received teriparatide, the major limitation of this study was the lack of placebo groups for comparison of the relative anti-osteoporosis efficacy among the subgroups of our patients. Another inherent limitation of this study is its post-hoc design. Since the high costs of TPTD made patients always refuse to fulfil the recommended course of 24 months, whereas Health Insurance in Qingdao only covers 12 months of expenses, the third limitation is that the study duration was relatively short; thus, the practical role of some factors might be concealed. Furthermore, other confounding factors must be investigated. Among several BTMs, only the serum PINP and β-CTX levels were evaluated. There is a probability that other BTMs might also be closely associated with LS BMD response. In addition, although an undesirable effect of baseline vitamin D deficiency was confirmed, the supplementary dosage schedule remains undetermined. As information regarding lifestyle elements was based on the participants’ self-reporting, there might be recall bias. Many efforts have been made to minimize this limitation. For instance, dietary and daily activity assessments were carried out using the IDQC and NZPAQ-SF, respectively, although the actual lifestyle of the participants might not have been captured perfectly. Therefore, future cohort studies would require more multi-institutional settings or randomized participant recruitment to validate the present findings.

### Supplementary Information

**Additional file 1.** 

## Data Availability

The datasets generated and/or analyzed during the current study are not publicly available due to property in copyright but are available from the corresponding author on reasonable request.
