# Supplementary material for: Analyzing the factors associated with efficacy among teriparatide treatment in postmenopausal women with osteoporosis
Source: BMC Musculoskelet Disord. 2024 Feb 3;25:109. doi: 10.1186/s12891-024-07227-1 (PMC10837964; doi:10.1186/s12891-024-07227-1)
Supplement: Supplementary file 1 — Additional file 1. [file 12891_2024_7227_MOESM1_ESM.docx]

Normal reference intervals for the serum markers involved.

| Variable | Reference interval | Unit of measurement |
| --- | --- | --- |
| 25(OH)VitD | ≥30 | ng/ml |
| PINP | 20.25-76.31 | ng/ml |
| β-CTX | 0-0.704 | ng/ml |
| AST | 7-40 | U/L |
| ALT | 13-35 | U/L |
| TG | ≤5.18 | mmol/L |
| UA | 155-357 | umol/L |
| Creatine | 35-81 | umol/L |
